# Supplementary figures and images for: Neural representation and modulation of volitional motivation in response to escalating efforts
Source: J Physiol. 2023 Jan 13;601(3):631–45. doi: 10.1113/JP283915 (PMC10108165; doi:10.1113/JP283915)

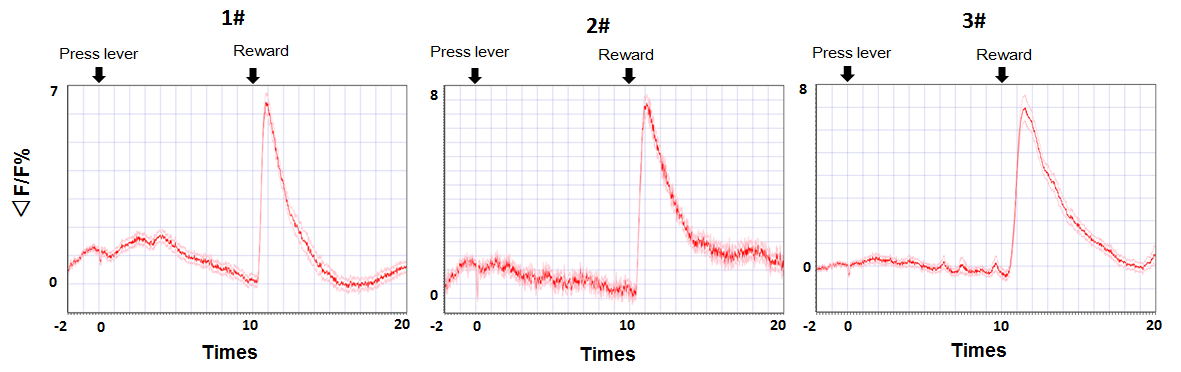

Supplement: Supplementary file 3 — Figure S1 [file TJP-601-631-s001.tif]
